# Characterization of the Major Histocompatibility Complex Class II Genes in Miiuy Croaker

**DOI:** 10.1371/journal.pone.0023823

**Published:** 2011-08-25

**Authors:** Tianjun Xu, Yuena Sun, Ge Shi, Yuanzhi Cheng, Rixin Wang

**Affiliations:** Laboratory for Marine Living Resources and Molecular Engineering, College of Marine Science, Zhejiang Ocean University, Zhoushan, People's Republic of China; Auburn University, United States of America

## Abstract

Major histocompatibility complex (MHC) has a central role in the adaptive immune system by presenting foreign peptide to the T-cell receptor. In order to study the molecular function and genomic characteristic of class II genes in teleost, the full lengths of MHC class IIA and IIB cDNA and genomic sequence were cloned from miiuy croaker (*Miichthys miiuy*). As in other teleost, four exons and three introns were identified in miiuy croaker class IIA gene; but the difference is that six exons and five introns were identified in the miiuy croaker class IIB gene. The deduced amino acid sequence of class IIA and class IIB had 26.3–85.7% and 11.0–88.8% identity with those of mammal and teleost, respectively. Real-time quantitative RT-PCR demonstrated that the MHC class IIA and IIB were ubiquitously expressed in ten normal tissues; expression levels of MHC genes were found first upregulated and then downregulated, and finally by a recovery to normal level throughout the pathogenic bacteria infection process. In addition, we report on the underlying mechanism that maintains sequences diversity among many fish species. A series of site-model tests implemented in the CODEML program revealed that positive Darwinian selection is likely the cause of the molecular evolution in the fish MHC class II genes.

## Introduction

The major histocompatibility complex (MHC) is a well-known and large genomic region in all vertebrates that encodes MHC molecules. MHC is central to the vertebrate immune system, its molecules that recognize and bind foreign peptides for presentation to specialist immune cells and subsequent initiation of an immune response [1]. The MHC it is recognized to be of importance for disease resistance, sexual selection and mate choice, therefore, they have been attracting attention of many scientists [2].

The MHC gene family includes two main subgroups of immunologically active molecules: class I and class II. Class II molecules are found on certain immune cells, chiefly antigen-presenting cells like macrophages, lymphocytes and dendritic cells, and present processed exogenous antigens to CD^4+^ T-helper cells [3], [4], which stimulate an immune reaction from other cells [2]. The MHC molecule comprises the peptide-binding region (PBR), which is responsible for antigen recognition, and a match between PBR and antigenic peptide is required to produce an immune cascade [2]. Class II molecule is a heterodimer that consists of an α and a β chain, which are encoded by two separate genes. The hallmark of MHC genes is the extremely high degree of polymorphism; polymorphism found in MHC class II genes is generally confined to exon 2, which encodes the PBR in the α1- and β1-domain. This diversity results in each individual being able to bind and present a variety of peptide ligands. Furthermore, diversity in MHC genes was found to be strongly associated with disease resistance in mammals and in non-mammalian species [5]–[10]. In general, positive selection leads to functional divergence of protein coding genes [11], [12]. Due to MHC molecules direct interaction with molecules of pathogens, these molecules are reported to have evolved adaptively, it is likely that much of these variation results from positive selection on the ability to recognize and present pathogens. Thus, the MHC genes may be an ideal choice to study adaptive molecular evolution in vertebrates.

The earliest research of associations between certain MHC alleles and disease resistance were inevitably focused on studies from a small number of important mammals, birds and aquaculture fish. It was highlighted that expanding studies including more species would clarify the immune mechanism and associations between MHC molecule and pathogens at MHC genes. The perceived problem was that the requirement to clone and characterize MHC genes in more species. Miiuy croaker, *Miichthys miiuy* is an important marine aquaculture fish that is mainly distributed from the western Japan to the East China Sea. Since it was bred in hatcheries successfully in the late 1990s, miiuy croaker has been widely cultured in China because of its commercial importance [13]. Many bacterial and parasite diseases especially had occurred at the stage of juvenile miiuy croaker, an important approach to disease prevention is to culture strains of fish with enhanced resistance to some major diseases using marker-assisted selection (MAS), and MHC genes are likely candidates as gene markers associated with disease resistance. In order to understand molecular polymorphism and the immune significance of MHC class II genes in this species, we report the identification of two MHC class II cDNA sequence and its gene organization. Gene expression analyses in different tissues and after *Vibrio anguillarum* injection were performed to elucidate the possible role of MHC molecules in the response against bacteria in miiuy croaker. Finally, to explore the ongoing evolutionary mechanisms of MHC genes in fish, studies of the molecular evolution of MHC genes are discussed.

## Materials and Methods

### Ethics statement

All work was conducted with the approval of the Animal Ethics Committee.

### Fish, challenge and sampling

Miiuy croakers (average weight, 750 g) were obtained from Zhoushan Fisheries Research Institute (Zhejiang, China). All fish were maintained in 1.5 m diameter tanks in a water recirculating system for one week prior to use in experiments to allow for acclimatization and evaluation of overall fish health. Only healthy fish, as determined by general appearance and level of activity, were used for the studies. Ten tissues (liver, spleen, kidney, intestines, heart, muscle, stomach, brain, swim bladder, and fin) of uninfected miiuy croaker were removed and kept at −80°C until use.

Challenge of miiuy croaker with pathogenic bacteria was performed as described by Xu et al. [14]. Fish were anaesthetized by immersion in MS222 and injected intraperitoneally with 1 ml bacteria suspension (3.0×10^7^ CFU/ml). Control fish injected with phosphate-buffered saline were maintained in separate tanks. The infected and health fish were killed at 6 h, 12 h, 24 h, 36 h, 48 h, and 72 h after injection, respectively. Tissues (liver, spleen, kidney, and intestine) were removed and kept at −80°C until use. Immediately following tissue excision, samples were placed into 1 mL of Trizol reagent and homogenised.

### DNA and RNA isolation, cDNA synthesis

Genomic DNA was extracted from fin samples of miiuy croaker with the method of phenol-chloroform. Total RNA was extracted from various tissues of adult individuals using Trizol reagent (Qiagen) according to the manufacture's instructions. Poly (A)^+^ RNAs were isolated from the total RNA using Oligotex™ spin-column kit (Qiagen). Complementary DNA was synthesized using BD Smart™ RACE cDNA amplification kit (Clontech) according to the manufacture's instructions.

### Primer design, amplification and cloning

In a preliminary study, we have identified the miiuy croaker MHC IIA and IIB EST sequences [14]. To isolate full length cDNA of MHC class II genes, four specific primers of two genes (GSP5' and GSP3' for each gene, respectively; Table S1, Supplementary Material online) were designed according to the candidate EST sequences. As such, to identify MHC class II genes genomic organization, primers (Table S1) were designed to amplify introns of MHC genes. Exon-intron junctions were deduced according to the known MHC class IIA and MHC IIB sequences of the other vertebrates. RACE-PCR was performed using a Smart RACE cDNA amplification kit (Clontech) according to the manufacturer's instructions. These PCR products were resolved by electrophoresis on 1% agarose gels and the fragments of interest were excised, and then purified using the Gel Extraction Kit (Takara). The purified fragments were ligated into pMD-19T vectors (Takara) and cloned to TOP10 cells according to the standard protocol. Positive clones were screened via PCR with M13+/- primers. At least three clones were sequenced per fragment using the ABI 3730xl automated sequencer with M13 primer.

### MHC genes expression

Primers MHC IIA-RT-F/MHC IIA-RT-R and MHC IIB-RT-F/MHC IIB-RT-R were used for amplifying MHC IIA and IIB fragment, respectively (Table S1). Real-time quantitative PCR was conducted on a 7500 Real-time PCR system (Applied Biosystems, USA). Expression of β-actin was used as internal control for MHC IIA and IIB gene expression analysis. The primers β-actin-RT-F and β-actin-RT-R (Table S1) were used for RT-PCR of β-actin expression.

### Sequence alignment and data analysis

Alignment of the nucleotide sequences and putative amino acid sequences of miiuy croaker and other known vertebrates were performed using MEGA 4.1 software [15]. Bayesian phylogenetic trees were constructed using MrBayes software [16]. The secondary structure was analyzed using PHDsec program. (EMBL).

### Test for selection

In order to estimate the selective constraints on the MHC class II genes, evolutionary analysis were performed with PAML v4 program suite [17], [18]. The hypothesis of positive selection was tested using site-specific model in the CODEML program. We employed the random-sites models [19] assuming several heterogeneous sites with different *ω* parameter without a priori knowledge of which class (neutral, purifying, or positive selection) a given codon belongs to. To account whether positive selection has been operating on any codon sites, we estimated parameters under six different codon substitution models (M0, M1a, M2a, M3, M7, and M8 model) [19]. The likelihood ratio tests (LRT) were performed to compare the corresponding models with and without selection (ie, M0 vs M3, M1a vs M2a, and M7 vs M8). When the alternative models M2a and M8 suggest the presence of codons with *ω*>1, this can be considered as evidence of positive selection [19]. Posterior probabilities for site classes have been calculated by Bays Empirical Bayes (BEB) in the case of models M2 and M8 [20]. Statistical significance is determined by comparing twice the log-likelihood scores (2ΔLnL) to a χ2 distribution with degrees equal to the difference in the number of parameters between the models to be compared [17]. The posterior means of *ω* for some sites classes are >1 (calculated are the average of *ω* over all sites classes weighted by the posterior probabilities), those sites are likely to be under positive selection [20].

## Results and Discussion

### Structure and genomic sequence of MHC IIA

The full-length cDNA of MHC class IIA gene designated as Mimi-DAA*0101 is 1257 bp, including 122 bp 5′ terminal untranslated region (UTR), 729 bp encoding region, 406 bp 3′ terminal UTR with a canonical polyadenylation signal (AATAAA) and a 18 bp poly (A) tail (GenBank accession No. GU936787). The 729 bp encoding region was found to code a protein with 242 amino acid residues.

The secondary structure of the putative MHC class IIA amino acid residues was analyzed via PHDsec program. One *N*-linked glycosylation site (N-X-S/T) and two protein kinase C phosphorylation site (S/T-X-R/K) were identified in the α2 domain region; four casein kinase II phosphorylation sites (S/T-X-X-D/E) were found in the α1 domain. In addition, six conserved cysteine residues among fishes were found in mature peptide domain, which are capable of forming the characteristic immunoglobulin domain disulfide bonds. The transmenbrane region of the miiuy croaker MHC class IIA molecule contains GxxxGxxGxxxG motif (where x is any hydrophobic residue other than Gly), it is important for correct interaction with the MHC class IIB chain [21].

Comparison of the deduced amino acid sequence of class IIA with other teleost and mammals showed that the general structure features of miiuy croaker MHC IIA are very similar to those presented in other species [22], [23]. The *N*-linked glycosylation site was found in α2 domain, however, it was not found in carp, gilthead sea bream, red sea bream and mammals. The length of transmenbrane domain of the miiuy croaker MHC IIA is same to those of other vertebrates. The GxxxGxxGxxxG motif of the miiuy croaker MHC IIA is shared by other fishes and mammals. The G residues of this motif involved in forming the face of α-helix that interacts with the corresponding phase of the β chain in the α-β dimmer, it is very important for correct interaction with the β chain [21].

**Figure 1:**
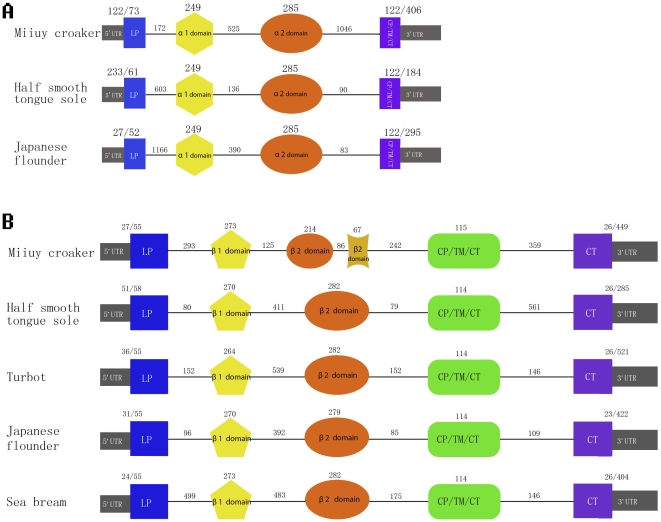
Schematic illustration of miiuy croaker class IIA gene (A) and IIB gene (B).

As in other vertebrates, four exons and three introns were identified in miiuy croaker. The genomic DNA containing the exon-intron structure resulting in a 3000 bp fragment is shown in Fig. S1, Supplementary Material online. Exon 1 includes a 122 bp 5′ terminal UTR and encodes the leader peptide followed by intron 1 of 172 bp, exon 2 encodes the α1 domain followed by intron 2 of 525 bp, exon 3 encodes the α2 domain followed by intron 3 of 1046 bp, exon 4 encodes the connecting peptide, the transmenbrane region, the cytoplasmic domain and includes 3′ terminal UTR. These genomic structural features of miiuy croaker are similar to those presented in other fishes [24]. Miiuy croaker class IIA has the longest intron 3 and leader peptide domain. However, mature peptide of miiuy croaker is of the same amino acid sequence length as that of other fishes (Fig. 1 (A)).

### Structure and genomic sequence of MHC IIB

The full length cDNA of MHC IIB gene designated as Mimi-DAB*0101 is 1226 bp, including 27 bp 5′ UTR, 750 bp encoding region, 449 bp 3′ UTR with an AATAAA motif and a 16 bp poly (A) tail (GenBank accession No. HM236158). The 750 bp encoding region was predicted to code a polypeptide with 249 amino acid residues.

Secondary structure analysis showed that two *N*-linked glycosylation sites were identified in the β1 domain region. Three protein kinase C phosphorylation sites and five casein kinase II phosphorylation sites were found in the β1, β2 and cytoplasmic domain, respectively. Four conserved cysteine residues were found in the β1 and β2 domain region of the amino acids sequence, which are capable of forming a couple of disulfide bonds. The transmenbrane region of the miiuy croaker MHC class IIB molecule contains GxxGxxxGxxxxxxG motif, which is believed to be important for correct interaction with the MHC IIA chain.

Comparison of the putative amino acid sequence of MHC class IIB with other fishes and mammalian revealed that the MHC class IIB consisted of two extracellular domains, a connecting peptide, a transmenbrane region and a cytoplasmic domain. Two *N*-linked glycosylation sites were found in the miiuy croaker while only one *N*-linked glycosylation site was found in other fishes. The additional *N*-linked glycosylation site (NST) was located in the miiuy croaker β1 domain, which was also found in mouse and human, but was absence in fishes such as carp, zebrafish, rainbow trout, Atlantic salmon and guppy. The numbers of *N*-linked glycosylation sites, protein kinase C phosphorylation sites, and casein kinase II phosphorylation sites were different from those presented in other fishes [24]. Existence of four conserved cysteine residues is to maintain the stability of protein structure of MHC class IIB molecule. The transmenbrane region of the miiuy croaker MHC class IIB molecule contains GxxGxxxGxxxxxxG motif, which is shared by half smooth tongue sole, flounder, European sea bass, red sea bass and trout.

The genomic structure resulting in a 2331 bp fragment is shown in Fig. S2, Supplementary Material online. Surprisingly, six exons and five introns were identified in the miiuy croaker class IIB gene (Fig. 1 (B)). However, only five exons and four introns were found in other fishes. Exon1 includes a 27 bp 5′ UTR and encodes the leader peptide, Exon1 and exon2 are separated by intron1 which is about 293 bp long. Exon 2 encodes the β1 domain followed by intron 2 of 125 bp. Exon 3 and exon 4 which encodes the β2 domain are separated by intron3 which is about 86 bp long. Exon 5-6 encodes the connecting peptide, the transmenbrane region, and the cytoplasmic domain and 3′UTR.

### Multiple alignment and phylogenetic analysis

**Figure 2:**
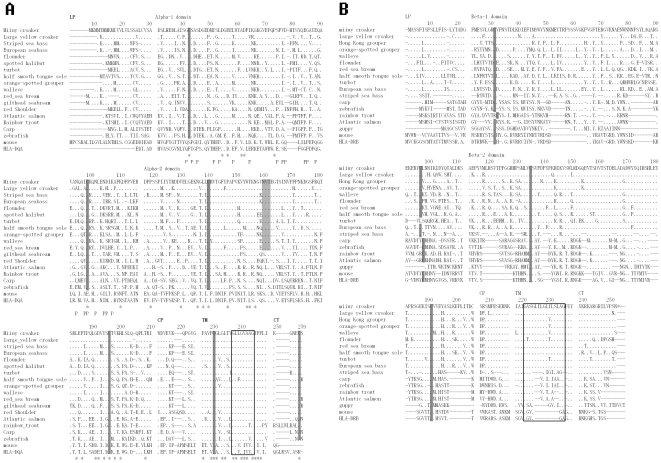
Alignment of deduced amino acid sequences of the miiuy croaker MHC class IIA (A) and IIB (B) gene with those of other teleost and mammalian. Identical amino acids are indicated by dots, and gaps used to maximize the alignment are shown by dashes, asterisks under the sequences denote mostly identical residues, “P” indicates the correlative amino acid that combines the peptide, conserved cysteins are shown in black background, and putative *N*-lined glycpsylation sites are shown in grey background. The box indicates CD8 binding loop; LP, leader peptide; CP, connecting peptide; TM, transmenbrane region; CT, cytoplasmic domain.

Multiple alignments of the deduced amino acid sequence of miiuy croaker MHC IIA and IIB gene and those of other vertebrates (Fig. 2) allowed for the characterization of functional domains. The miiuy croaker MHC IIA and IIB gene shares many of the same characteristics as other fishes, such as the conserved cysteines that provide molecular stability, and GxxxGxxGxxxG motif or GxxGxxxGxxxxxxG motif, which is believed to be important for correct interaction with each other in MHC II molecule. At the amino acid level, class IIA and IIB sequences have high identification with those of other teleost, but an extensive variability was detected in the α1 and β1 domain, which corresponds with the functional peptide-binding region of MHC class II molecules.

**Figure 3:**
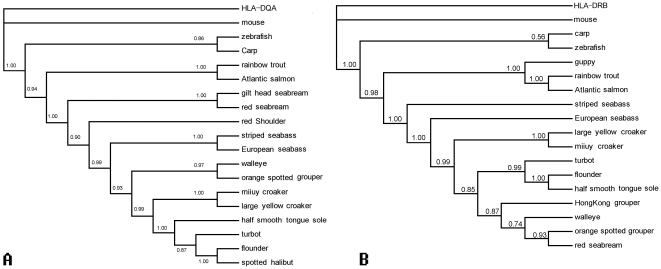
Phylogenetic tree of MHC class IIA (A) and IIB (B) gene from miiuy croaker and other vertebrates were constructed using MrBayes with the Bayesian method.

To study the phylogenetic relationship of miiuy croaker MHC IIA and IIB gene and that of the other vertebrate species, two dendrograms were constructed using the Bayesian method (Fig. 3). Phylogenetic analysis demonstrated that the deduced amino acid sequence of miiuy croaker MHC IIA (*Mimi*-DAA*0101) had 85.7, 76.1, 76.1, 74.9, 73.4, 70.3, 68.7, 67.2, 67.2, 66.0, 64.5, 62.5, 56.4, 53.7, 51.0, 46.3, 27.0 and 26.3% identities to those of large yellow croaker (EF681861), striped sea bass (AAB67867), European sea bass (DQ821106), orange-spotted grouper (FJ598317), walleye (AY158872), gilthead seabream (DQ019411), spotted halibut (GU253882), red sea bream (AAW21980), flounder (AY997530), turbot (DQ094170), half smooth tongue sole (FJ372721), red shoulder (AF212850), Atlantic salmon (AAL40122), rainbow trout (CAB96452), zebrafish (CAD60677), carp (CAA64707), mouse (BAE42123), and human (HLA-DQA, AAC41950) respectively. Phylogenetic tree showed that the miiuy croaker clustered together with large yellow croaker, both of which belong to the same family.

Similar results were obtained from the alignment of the deduced amino acid sequence of miiuy croaker MHC IIB. The dendrogram showed that class IIB (*Mimi*-DAB*0101) shared 88.8, 80.7, 77.6, 76.4, 74.4, 74.0, 69.3, 68.1, 66.5, 48.0, 31.5, 30.7, 26.4, 26.0, 20.1, 13.0, and 11.0% identity to those of large yellow croaker (EF681865), Hong Kong grouper (EU399187), orange-spotted grouper (FJ598318), European sea bass (DQ821113), red sea bream (AY190711), walleye (AY158837), turbot (DQ001730), flounder (AY848955), half smooth tongue sole (FJ372722), striped sea bass (AAA49379), carp (AA64706), zebrafish (CAD87794), rainbow trout (AF115529), Atlantic salmon (CAA49726), guppy (AF080585), human (HLA-DRB, M11161) and mouse (P18469). Miiuy croaker and large yellow croaker are also clustered together in phylogenetic tree.

### Expression analysis after bacterial infection

**Figure 4:**
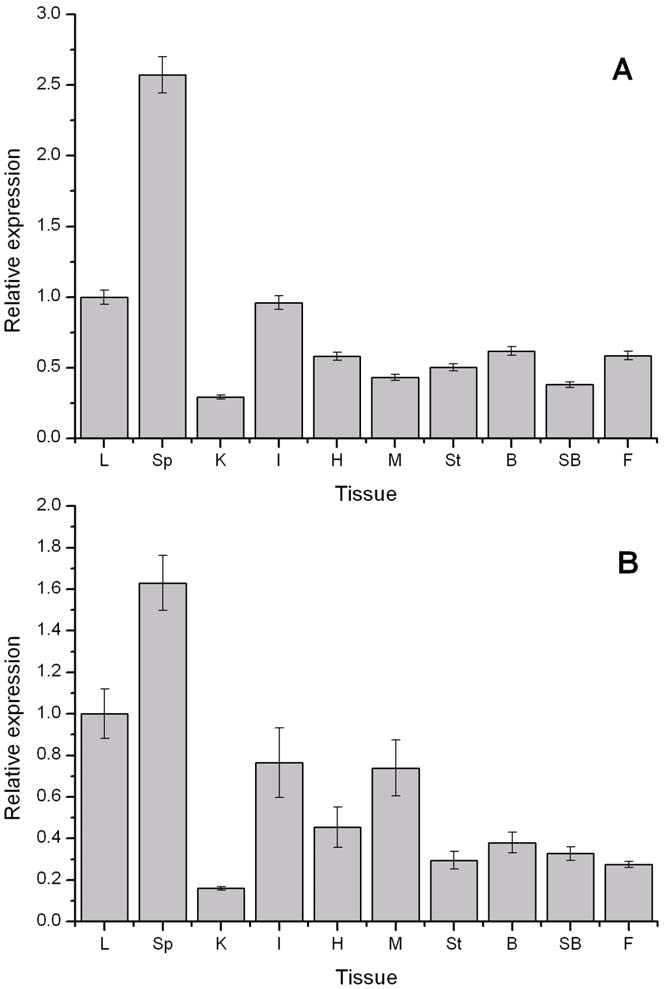
Expression of MHC IIA (A) and IIB (B) gene in various tissues (liver (L), spleen (Sp), kidney (K), intestines (I), heart (H), muscle (M), stomach (St), brain (B), Swim bladder (SB), and fin (F) of uninfected miiuy croaker. MHC gene mRNA levels were expressed as a relative ratio to beta-actin levels in the same samples after real-time PCR.

Real-time quantitative RT-PCR demonstrated that MHC genes were ubiquitously expressed in ten tissues, but the expression level was distinctly different. Expression results showed that high levels of transcripts of MHC IIA and IIB were detected in spleen, liver and intestines, moderate levels in heart, muscle, stomach, brain, swim bladder and fin, and low expression in kidney (Fig. 4). Generally, MHC II genes expressed on dendritic cells, B cells and macrophages in mammals [25]. But different conclusions were found in different fishes, such as, low expression level in brain and skeletal muscle of MHC class IIB were found in Atlantic salmon. However, Rodrigues et al. [26] detected no expression of class IIB gene in carp heart, muscle. In the present study, expression of MHC genes of miiuy croaker showed that MHC class IIA and IIB genes expressed in a ubiquitous manner with different levels in different tissues, this expression pattern is similar to that in mammals. The high expression in spleen, liver and intestines is similar to that in other fish species [3], [24], which may imply that the fish immune response occurs through these immune organs when pathogenic antigens enter the fish, and where the fish immune response is initiated.

**Figure 5:**
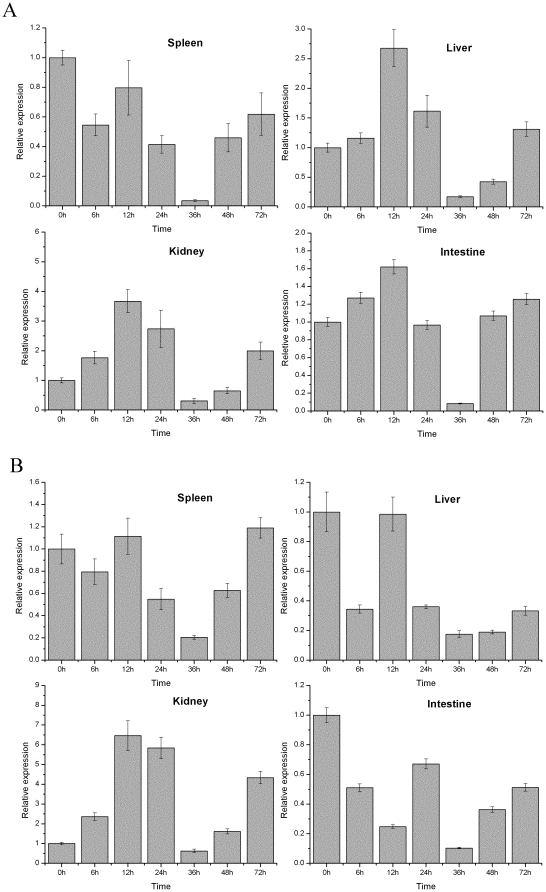
Expression of MHC IIA (A) and IIB (B) gene in four tissues after injection with *V. anguillarum* sampled at 6, 12, 24, 36, 48, and 72 h after injection.

Challenge of miiuy croaker with the pathogenic bacteria, *V. anguillarum*, resulted in significant changes in the expression of MHC IIA and IIB mRNA from infection starting time to 72 h after challenge in four immune organs (Fig. 5). In kidney, liver and intestines, the expression level of MHC IIA mRNA constantly increased from infection starting time to 12h, the highest expression level were occurred at 12 h after pathogenic bacteria injection; and then sustained decreased from 12 h to 36 h, the lowest expression level were checked at 36h after infection; followed by a recovery to normal level after 72h in all four organs. The same approach to study the expression of MHC IIB mRNA after infection showed similar results, overview, the expression levels of MHC IIB mRNA first increased and then decreased, and finally return to normal levels, expression levels of highest and lowest values were also found at the 12h and 36h. In the intestines, the highest expression level was found at 24h after injection. Expression levels of MHC genes were found first upregulated and then downregulated, and finally recovery to normal level throughout the infection process suggest that crucial interference of cellular function occurs under a semilethal concentration of pathogenic bacteria in immune tissues. If the infected concentration of pathogenic bacteria is greater than similethal, the cellular function of immunity organs may be destroyed, and the corresponding expression may decrease [7]. Significant changes of MHC genes occurred in spleen, liver, kidney and intestines, which may be important and sensitive organs for MHC II molecule function.

### Patterns of positive selection

To explore the ongoing evolutionary mechanisms of MHC class II genes in fish, random sites models of PAML were used to detect positive selection [18], [27]–[29]. In this study, MHC IIA sequences group and MHC IIB sequences group were used for molecular evolution analyses, respectively,. The random sites models demonstrate extreme variability in selective pressure among sites and the presence of a number of sites under positive selection. In MHC IIA group, model M3 appears to be a better fit to the data than the M0 model, suggesting variable selection pressure across the MHC IIA sequences, and these alternative hypothesis models were significantly better than the null hypothesis models (M2 vs M1, *p* = 0; M8 vs M7, *p* = 0) (Table 1). The site-specific analysis in PAML identified positively selected sites in the MHC IIA group (Fig. S3, Supplementary Material online; Table 1). Model M2 and M8 revealed the same nine codon sites with posterior probability >0.95. In addition, in MHC IIB group, LRTs revealed that models which allowed for adaptive selection fitted the data better than those which did not (M2 vs M1, *p* = 0; M8 vs M7, *p* = 0) (Table 1). The site-specific analysis in PAML identified many positively selected sites in the MHC IIB group (Fig. S4, Supplementary Material online; Table 1). Thirteen codon sites were significantly positively selected detected by model M2 and M8, respectively, with the posterior probability >0.95 under adaptive selection model. Model M8 in PAML using BEB, which is more suited for smaller data sets [20], should therefore be considered the most reliable result. In Fig. S3 and Fig. S4, we can see that seven condons with posterior probability >0.95 (78% out of all positive selection sites with posterior probability >0.95) were appeared in the α1 domain; and thirteen condons (100% out of all of all positive selection sites with posterior probability >0.95) were detected in the β1 domain. The hallmark of MHC genes is the high degree of polymorphism; in generally, high polymorphism found in MHC class II genes is confined to the α1- and β1-domain that encode the peptide binding region. This diversity results in individual being able to bind and present a variety of peptide ligands, much of these variations derived from positive selection on the ability to recognize and present pathogens. Our results in this study revealed that the observed diversity in the MHC class II gene may be to a large extent generated by positive selection on PBR sites.

**Table 1:** Evidence of positive Darwinian selection from site-specific model analyse for the MHC class II genes of fish

Groups	Model	*P*	Log-li*k*elihood	Model comparison	Parameter estimates	Positively selected sites
Class IIA (DAA)	M0 (One ration)	1	-5529.065		*K* = 1.362,*ω* = 0.595	NA
	M1a (Nearly Neutral)	1	-5304.989		*K* = 1.304, *p*_0_ = 0.555, *p*_1_ = 0. 445,*ω*_0_ = 0.093, *ω*_1_ = 1.0	NA
	M2a (Positive Selection)	3	-5243.361	M2a vs M1a, 2ΔLnL = 123.26, df = 2, *p* = 0.00	*K* = 1.429, *p*_0_ = 0.514, *p*_1_ = 0.399, *p*_2_ = 0.087,*ω*_0_ = 0.099, *ω*_1_ = 1.000,***ω*** **_2_ = 4.298**	**20,22,** *31,* **40,** *56,*62,63,**65,68,71,81,** *85,* **86,**87,*95,* **97,**139,180, *185,*197
	M3 (discrete)	5	-5242.862	M3 vs M0, 2ΔLnL = 572.41, df = 4, *p* = 0.00	*K* = 1.449, *p*_0_ = 0.529, *p*_1_ = 0.392, *p*_2_ = 0.079,*ω*_0_ = 0.110, *ω*_1_ = 1.120,***ω*** **_2_ = 4.721**	Not allowed
	M7 (beta)	2	-5312.996		*K* = 1.266, *p* = 0.273, *q* = 0.324	NA
	M8 (beta and omega)	4	-5248.963	M8 vs M7, 2ΔLnL = 128.01, df = 2, *p* = 0.00	*K* = 1.404, *p*_0_ = 0.905, *p*_1_ = 0.095, *p* = 0.308, *q* = 0.386,***ω*** ** = 3.845**	**20,22,** *31,* **40,**56,62,63,**65,68,71,81,** *85,* **86,**87,95,**97,**139,180,185
Class IIB (DAB)	M0 (One ration)	1	-5223.590		*K* = 1.668,*ω* = 0.426	NA
	M1a (Nearly Neutral)	1	-4998.839		*K* = 1.657, *p*_0_ = 0.649, *p*_1_ = 0.351,*ω*_0_ = 0.106, *ω*_1_ = 1.0	NA
	M2a (Positive Selection)	3	-4946.438	M2a vs M1a, 2ΔLnL = 104.80, df = 2, *p* = 0.00	*K* = 1.853, *p*_0_ = 0.603, *p*_1_ = 0.324, *p*_2_ = 0.073,*ω*_0_ = 0.115, *ω*_1_ = 1,***ω*** **_2_ = 7.507**	**25,26,28,** 29, **30,46,48,**55,**71,78,**81,88,89,,**98,103,104,106,107,**150
	M3 (discrete)	5	-4946.358	M3 vs M0, 2ΔLnL = 554.46, df = 4, *p* = 0.00	*K* = 1.849, *p*_0_ = 0.586, *p*_1_ = 0.330, *p*_2_ = 0.084,*ω*_0_ = 0.108*ω*_1_ = 0.907, ***ω*** **_2_ = 6.141**	Not allowed
	M7 (beta)	2	-5013.622		*K* = 1.607, *p* = 0.372, *q* = 0.581	NA
	M8 (beta and omega)	4	-4951.518	M8 vs M7, 2ΔLnL = 124.21, df = 2, *p* = 0.00	*K* = 1.725, *p*_0_ = 0.897, *p*_1_ = 0.103, *p* = 0.514, *q* = 0.999,***ω*** ** = 4.599**	**25,26,28,** *29,* **30,46,48,**55,**71,78,**81,88,89,**98,103,104,106,107,**150

Note: *P* number of parameters in the *ω* distribution, *K* estimated transition/transversion rate ration, *ω* selection parameter, and *p*_n_ proportion of sites that fall into the *ω_n_* site class. *p*, *q* shape parameters of the β function (for models M7 and M8). Positively selected sites with posterior probability >0.95 are in bold, 0.9–0.95 are underlined, 0.8–0.9 are italics, and 0.5–0.8 in plain text.

## Supporting Information

Figure S1Genomic sequence of miiuy croaker class IIA gene. Exons are in uppercase and introns are in lowercase. The stop codon is indicated by an asterisk. *N*-linked glycosylation site are represented with boxes; protein kinase C phosphorylation sites are underlined with
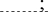
casein kinase II phosphorylation sites are underlined with 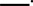

(TIF)

Figure S2Genomic sequence of miiuy croaker class IIB gene. Exons are in uppercase and introns are in lowercase. The stop codon is indicated by an asterisk. *N*-linked glycosylation site are represented with boxes; protein kinase C phosphorylation sites are underlined with
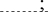
casein kinase II phosphorylation sites are underlined with 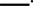

(TIF)

Figure S3 Amino acid sequence comparison among fish MHC IIA sequences. Positively selected sites identified using M8 model (Table 1) are shaded in black background.
(TIF)

Figure S4 Amino acid sequence comparison among fish MHC IIB sequences. Positively selected sites identified using M8 model (Table 1) are shaded in black background.
(TIF)

Table S1 Primers used in this study.
(DOC)

## References

[1] Klein J (1986). Natural history of the major histocompatibility complex.. 1st edn.

[2] Piertney SB, Oliver MK (2006). The evolutionary ecology of the major histocompatibility complex.. Heredity.

[3] Srisapoome P, Ohira T, Hirono I, Aoki T (2004). Cloning, characterization and expression of cDNA containing major histocompatibility complex class IIα and IIβ genes of Japanese flounder *Paralichthys olivaceus*.. Fisheries Science.

[4] Kjøglum S, Larsen S, Bakke HG, Grimholt U (2006). How specific MHC class I and class II combinations affect disease resistance against infectious salmon anaemia in Atlantic salmon (*Salmo salar*).. Fish Shellfish Immunol.

[5] Bacon LD (1987). Influence of the major histocompatibility complex on disease resistance and productivity.. Poultry Science.

[6] Langefors A, Lohm J, Grahn M, Andersen O, von Schantz T (2001). Association between major histocompatibility complex class IIB alleles and resistance to *Aeromonas salmonicida* in Atlantic salmon.. Proc Biol Sci.

[7] Zhang YX, Chen SL, Liu YG, Sha ZX, Liu ZJ (2006). Major histocompatibility complex II B allele polymorphism and its association with resistance/susceptibility to *Vibrio anguillarum* in Japanese Flounder (*Paralichthys olivaceus*).. Marine Biothchnology.

[8] Glover KA, Grimholt U, Bakke HG, Nilsen F, Storset A (2007). Major histocompatibility complex (MHC) variation and susceptibility to the sea louse lepeophtheirus salmonis in Atlantic salmon *Salmo Salar*.. Diseases of Aquatic Organisms.

[9] Wynne JW, Cook MT, Nowak BF, Elliott NG (2007). Major histocompatibility polymorphism associated with resistance towards amoebic gill disease in Atlantic salmon (*Salmo salar* L.).. Fish Shellfish Immunol.

[10] Xu TJ, Chen SL, Ji XS, Tian YS (2008). MHC polymorphism and disease resistance to vivrio anguillarum in 12 selective Japanese flounder (*Paralichthys olivaceus*) families.. Fish Shellfish Immunol.

[11] Kimura M (1983). The neutral theory of molecular evolution..

[12] Ford M (2002). Applications of selective neutrality tests to molecular ecology.. Mol Ecol.

[13] Lou B (2004). Biology and breeding technology of Miichthys miiuy.. Chin J Mod Fish..

[14] Xu TJ, Meng FX, Sun YN, Shi G, Wang RX (2010). Identification of immune genes of the miiuy croaker (*Miichthys miiuy*) by sequencing and bioinformatic analysis of ESTs.. Fish Shellfish Immunol.

[15] Tamura K, Dudley J, Nei M, Kumar S (2007). MEGA4: Molecular Evolutionary Genetics Analysis (MEGA) Software Version 4.0.. Mol Biol Evol.

[16] Ronquist F, Huelsenbeck JP (2003). MrBayes 3: Bayesian phylogenetic inference under mixed models.. Bioinformatics.

[17] Yang Z (1997). PAML: a program package for phylogenetic analysis by maximum likelihood.. Comput Appl Biosci.

[18] Yang Z (2007). PAML 4: phylogenetic analysis by maximum likelihood.. Mol Biol Evol.

[19] Yang Z, Nielsen R, Goldman N, Pedersen AMK (2000). Codon substitution models for heterogeneous selection pressure at amino acid sites.. Genetics.

[20] Yang Z, Wong WSW, Nielsen R (2005). Bayes Empirical Bayes inference of amino acid sites under positive selection.. Mol Biol Evol.

[21] Cosson P, Bonifacino JS (1992). Role of transmembrane domain interactions in the assembly of class II MHC molecules.. Science.

[22] Zhang YX, Chen SL (2006). Molecular identification, polymorphism and expression analysis of major histocompatibility complex class II A and B genes of turbot (*Scophthalmus maximus*).. Marine Biotechnology.

[23] Grimholt U, Getahum A, Hermsen T, Stet RJ (2000). The major histocompatibility complex class II alpha chain in salmonid fishes.. Dev Comp Immunol.

[24] Xu TJ, Chen SL, Ji XS, Sha ZX (2009). Molecular cloning, genomic structure, polymorphism and expression analysis of major histocompatibility complex class IIA and IIB genes of half-smooth tongue sole (*Cynoglossus semilaevis*).. Fish&Shellfish Immunology.

[25] Dixon B, van Erp SH, Rodrigues PN, Egberts, Stet RJ (1995). Fish major histocompatibility complex genes, an expansion.. Dev Comp Immunol.

[26] Rodrigues PN, Hermsen TT, Rombout JH, Egberts E, Stet RJ (1995). Detection of MHC class II transcripts in lymphoid tissues of the common carp (*Crprinus carpio* L).. Dev Comp Immunol.

[27] Padhi A, Verghese B (2007). Evidence for positive Darwinian selection on the hepcidin gene of Perciform and Pleuronectiform fishes.. Mol Divers.

[28] Chen JS, Wang T, Tzeng T, Wang C, Wang D (2008). Evidence for positive selection in the TLR9 gene of teleosts.. Fish Shellfish Immunol.

[29] Fernandes JMO, Ruangsri J, Kiron V (2010). Atlantic Cod Piscidin and Its Diversification through Positive Selection.. Plos one.

