# Supplementary figures and images for: Characterization of the Major Histocompatibility Complex Class II Genes in Miiuy Croaker
Source: PLoS One. 2011 Aug 25;6(8):e23823. doi: 10.1371/journal.pone.0023823 (PMC3162010; doi:10.1371/journal.pone.0023823)

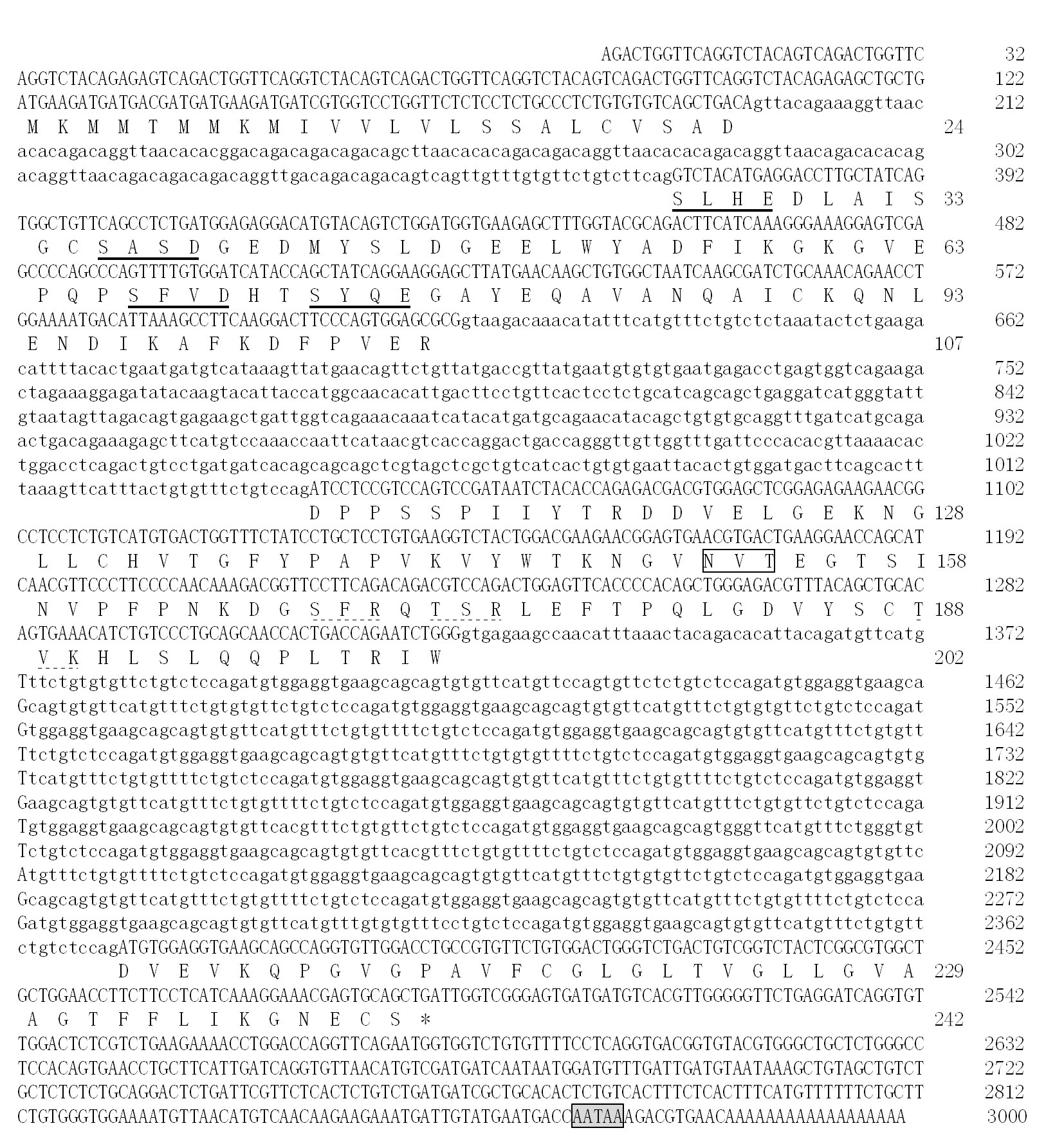

Supplement: Figure S1 — Genomic sequence of miiuy croaker class IIA gene. Exons are in uppercase and introns are in lowercase. The stop codon is indicated by an asterisk. N-linked glycosylation site are represented with boxes; protein kinase C phosphorylation sites are underlined withcasein kinase II phosphorylation sites are underlined with (TIF) [file pone.0023823.s001.tif]

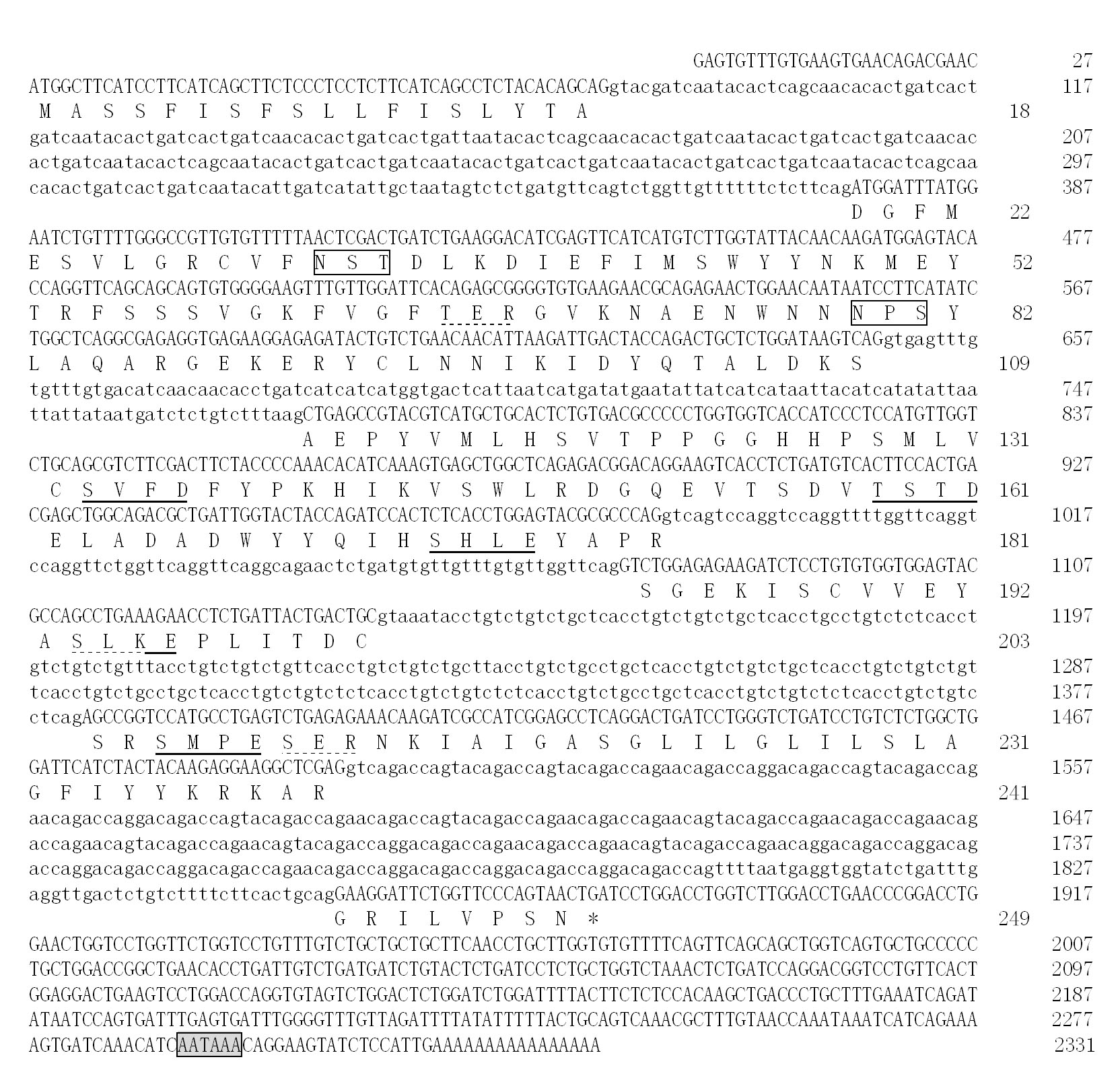

Supplement: Figure S2 — Genomic sequence of miiuy croaker class IIB gene. Exons are in uppercase and introns are in lowercase. The stop codon is indicated by an asterisk. N-linked glycosylation site are represented with boxes; protein kinase C phosphorylation sites are underlined withcasein kinase II phosphorylation sites are underlined with (TIF) [file pone.0023823.s002.tif]

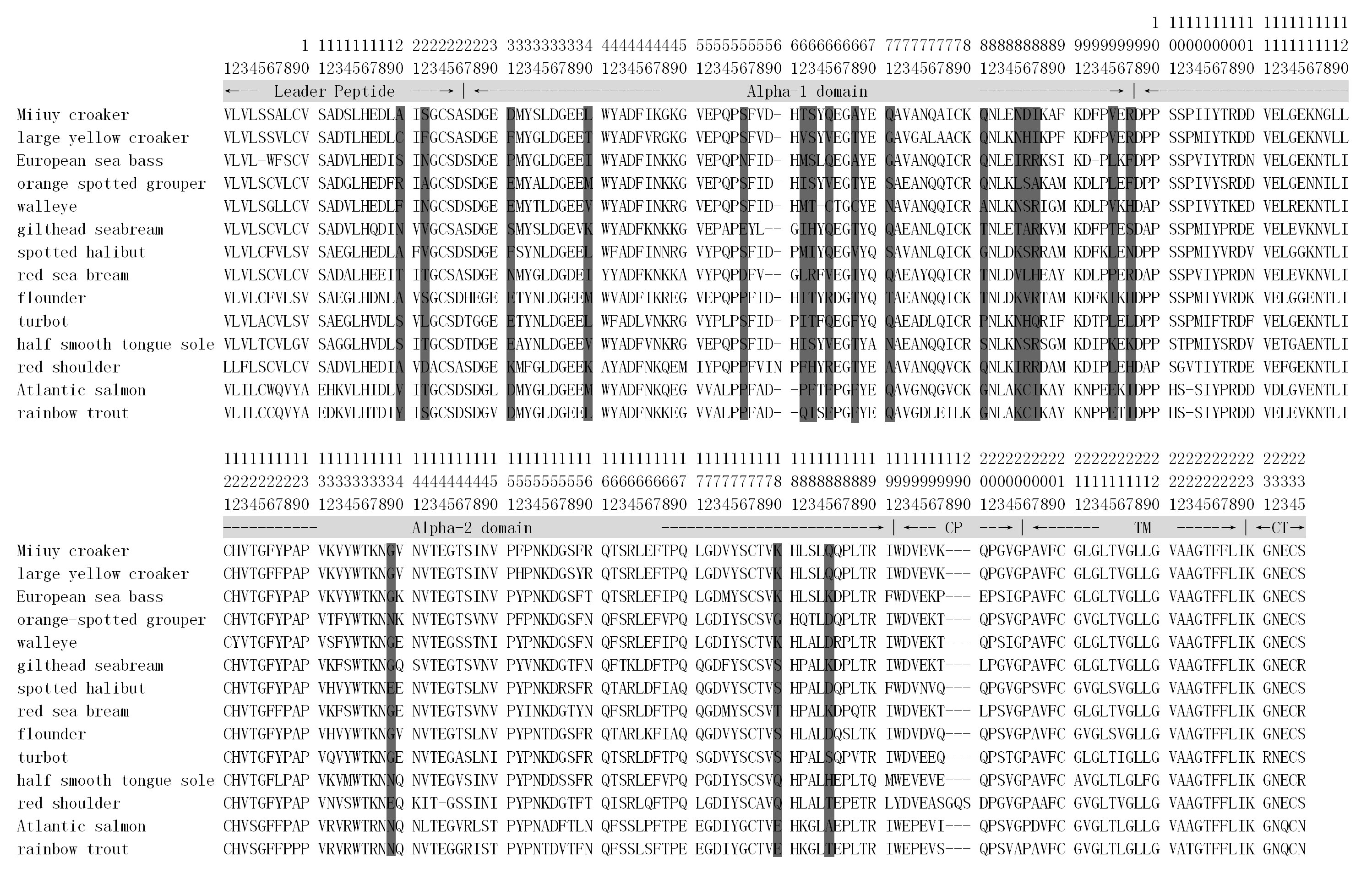

Supplement: Figure S3 — Amino acid sequence comparison among fish MHC IIA sequences. Positively selected sites identified using M8 model (Table 1) are shaded in black background. (TIF) [file pone.0023823.s003.tif]

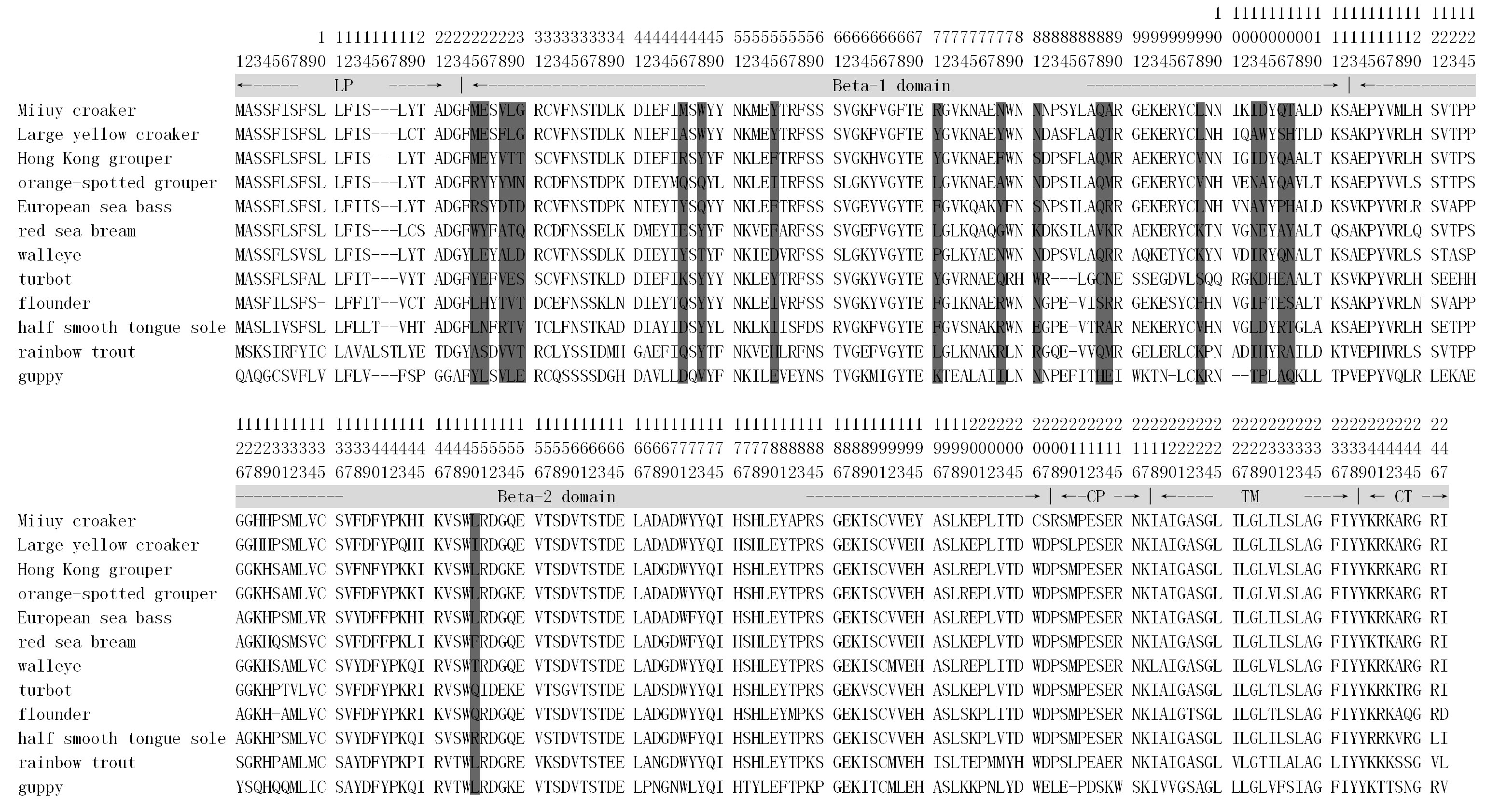

Supplement: Figure S4 — Amino acid sequence comparison among fish MHC IIB sequences. Positively selected sites identified using M8 model (Table 1) are shaded in black background. (TIF) [file pone.0023823.s004.tif]
