# Supplementary material for: Characterization of the Major Histocompatibility Complex Class II Genes in Miiuy Croaker
Source: PLoS One. 2011 Aug 25;6(8):e23823. doi: 10.1371/journal.pone.0023823 (PMC3162010; doi:10.1371/journal.pone.0023823)
Supplement: Table S1 — Primers used in this study. (DOC) [file pone.0023823.s005.doc]

**Table S1** Primers used in this study

| Name | Primers sequences (5’-3’) | Amplification target |
| --- | --- | --- |
| MHC IIA-GSP5’ | GGTGTAGATTATCGGACTGGACGGAGG | For 5’RACE of MHC IIA |
| MHC IIA-GSP3’ | CCTCCGTCCAGTCCGATAATCTACACC | For 3’RACE of MHC IIA |
| MHC IIB-GSP5’ | TGAATCCAACAAACTTCCCCACACTGC | For 5’RACE of MHC IIB |
| MHC IIB-GSP3’ | AGGTTCAGCAGCAGTGTGGGGAAGTTT | For 3’RACE of MHC IIB |
| MHC IIA-RT-F | CATCAAAGGGAAAGGAGT | Expression of MHC IIA |
| MHC IIA-RT-R | AGGAGCAGGATAGAAACC |
| MHC IIB-RT-F | ACAAGATCGCCATCGGAGC | Expression of MHC IIB |
| MHC IIB-RT-R | CACCAAGCAGGTTGAAGCA |
| β-actin-RT-F | GTGATGAAGCCCAGAGCA | Expression of β-actin |
| β-actin-RT-R | CGACCAGAGGCATACAGG |
| MHC IIA-intron-1F | ATGATCGTGGTCCTGGTT | Intron 1 of MHC IIA |
| MHC IIA-intron-1R | CTCGACTCCTTTCCCTTT |
| MHC IIA-intron-2F | TTCATCAAAGGGAAAGGAGT | Intron 2 of MHC IIA |
| MHC IIA-intron-2R | CTCTGGTGTAGATTATCGGACT |
| MHC IIA-intron-3F | TCCAGTCCGATAATCTACAC | Intron 3 of MHC IIA |
| MHC IIA-intron-3R | GTCAGACCCAGTCCACAG |
| MHC IIB-intron-1F | CATCAGCTTCTCCCTCCT | Intron 1 of MHC IIB |
| MHC IIB-intron-1R | AACCTGGTGTACTCCATCTTGT |
| MHC IIB-intron-2F | ACCAGGTTCAGCAGCAGT | Intron 2 of MHC IIB |
| MHC IIB-intron-2R | AGACCAACATGGAGGGAT |
| MHC IIB-intron-3F | GCGTCTTCGACTTCTACC | Intron 3 and 4 of MHC IIB |
| MHC IIB-intron-3R | TCAGACTCAGGCATGGAC |
| MHC IIB-intron-4F | GGCTGGATTCATCTACTACAA | Intron 5 of MHC IIB |
| MHC IIB-intron-4R | GATTTCAAAGCAGGGTCA |
